# Supplementary material for: GLUT3/SLC2A3 Is an Endogenous Marker of Hypoxia in Prostate Cancer Cell Lines and Patient-Derived Xenograft Tumors
Source: Diagnostics (Basel). 2022 Mar 10;12(3):676. doi: 10.3390/diagnostics12030676 (PMC8946944; doi:10.3390/diagnostics12030676)
Supplement: Supplementary file 1 [file diagnostics-12-00676-s001.zip › Supplementary figures.pdf]

Supplementary Figure S1

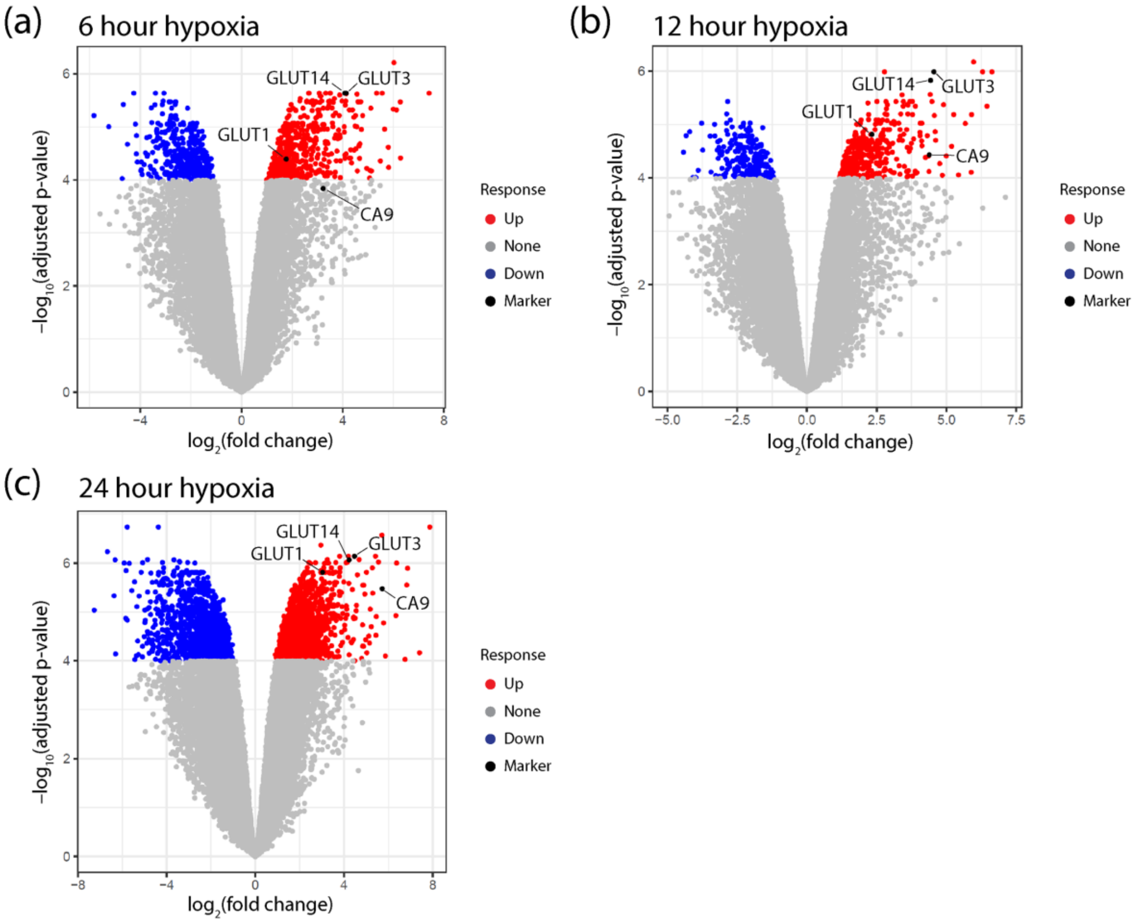

Figure S1. Volcano plots of RNA-seq data from hypoxic (1% O_2) RWPE1 cells at (**a**) 6, (**b**) 12, and (**c**) 24 hour time points. Plots show the log_2FC over each time point relative to 0 hour normoxia samples (x-axis), and significance (computed as $-log_{10}P_{adj}$) over each time point relative to 0 hour normoxia samples (y-axis).

Supplementary Figure S2

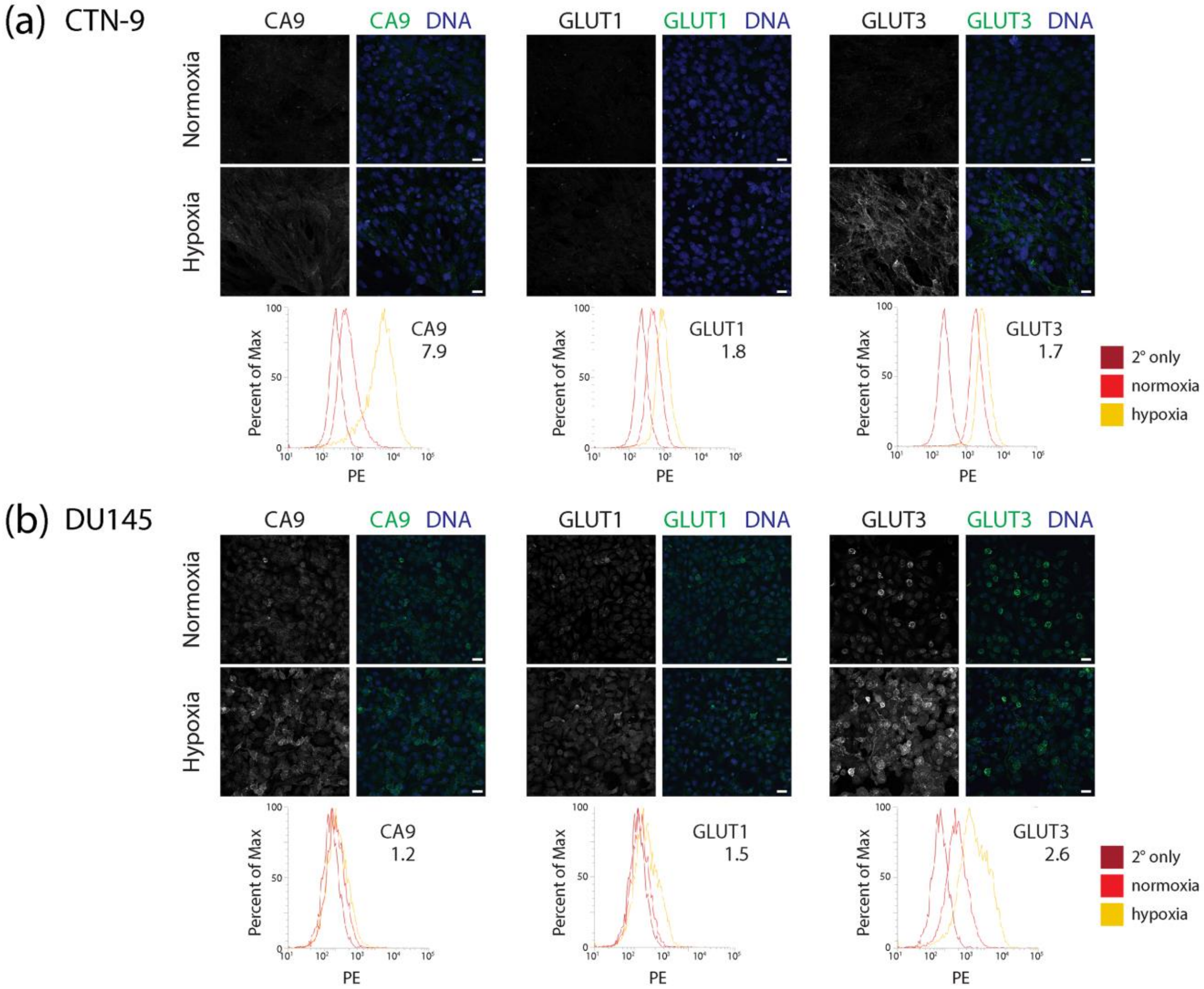

Figure S2. Hypoxia-mediated upregulation of GLUT3 is observed in transformed prostate cells and an established prostate cancer cell line. (**a**) CTN-9 and (**b**) DU145 cells were cultured in either normoxic or hypoxic (1% O_2) conditions for 48 hours and then processed for immunofluorescence microscopy (upper and middle panels) or flow cytometry (lower panels). CTN-9 is a transformed line derived from PrEC-Hahn cells [52]. Cells were stained for either CA9, GLUT1, or GLUT3 (green, upper and middle panels). DNA, blue. Scale, 25 μm. (Lower panels) Comparison of flow cytometry profiles of CA9, GLUT1, or GLUT3 in cells cultured in normoxia (red) versus hypoxia (yellow). Cells stained with only secondary antibody is shown (dark red). The fold increase in level of the indicated proteins is denoted. PE, phycoerythrin.

Supplementary Figure S3

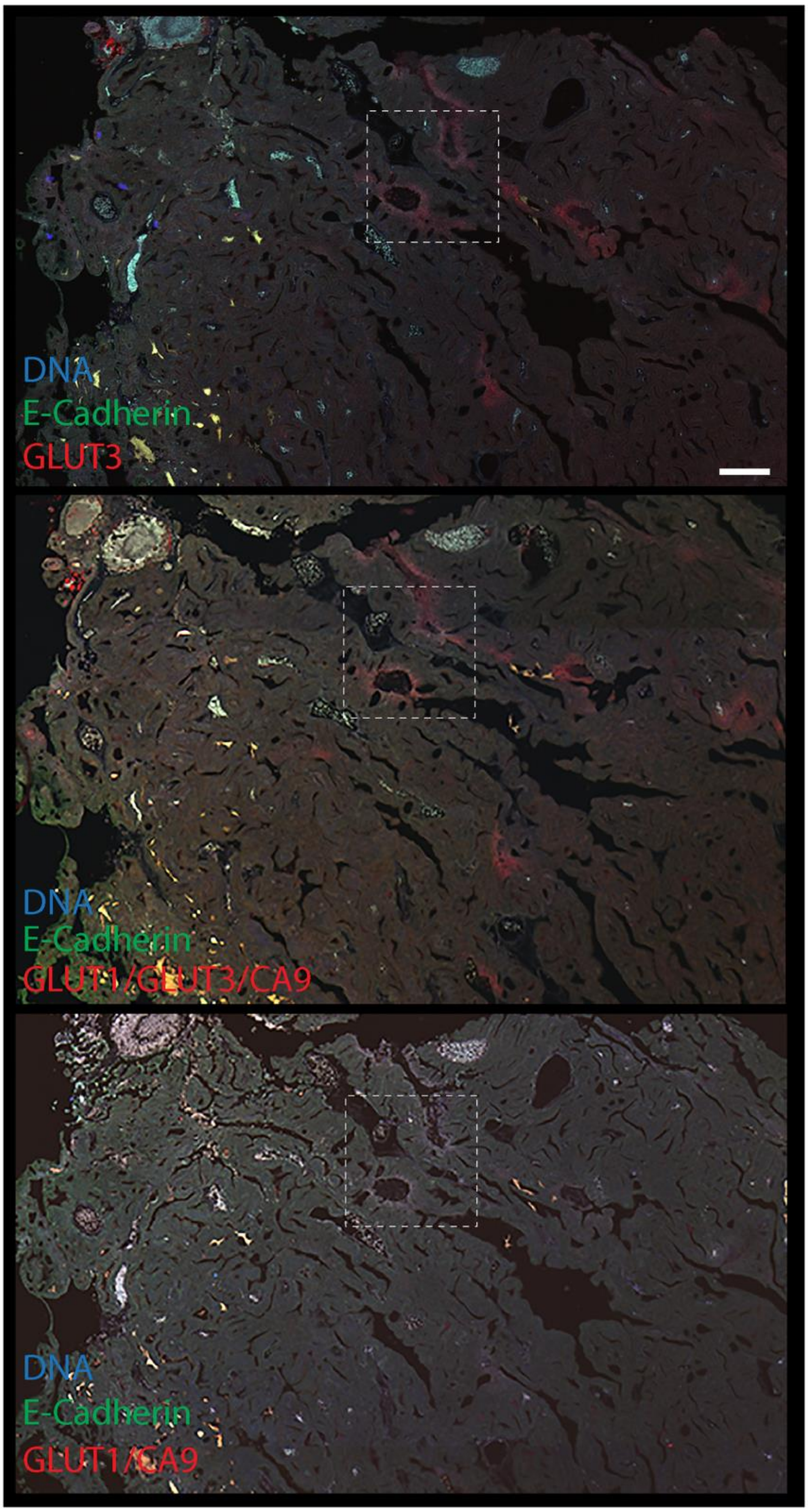

Figure S3. Patient-derived xenograph (PDX) model of primary prostate cancer contains distinct pockets of GLUT3 staining that do not overlap with GLUT1 or CA9. Adjacent sections of PDX tumor immunostained for E-Cadherin (green) to mark cell borders and GLUT3 (red, upper panel), a cocktail containing anti-GLUT1 and anti-CA9 antibodes (red, lower panel), or antibodies against all three proteins GLUT1, GLUT3, and CA9 (red, middle panel) as indicated. DNA, blue. Boxed region is shown at higher magnification in Figure 6c. Scale, 250 μm. .
